# Broadband multiple responses of surface modes in quasicrystalline plasmonic structure

**DOI:** 10.1038/srep30818

**Published:** 2016-08-05

**Authors:** Haiming Yuan, Xiangqian Jiang, Feng Huang, Xiudong Sun

**Affiliations:** 1 Department of Physics, Harbin Institute of Technology, Harbin 150001, China; 2 Key Lab of Micro-Optics and Photonic Technology of Heilongjiang Province, Harbin 150001, China

## Abstract

We numerically study the multiple excitation of surface modes in 2D photonic quasicrystal/metal/substrate structure. An improved rigorous coupled wave analysis method that can handle the quasicrystalline structure is presented. The quasicrystalline lattice, which refers to Penrose tiling in this paper, is generated by the cut-and-project method. The normal incidence spectrum presents a broadband multiple responses property. We find that the phase matching condition determines the excitation frequency for a given incident angle, while the depth of the reflection valley depends on the incident polarization. The modes will split into several sub-modes at oblique incidence, which give rise to the appearance of more responses on the spectrum.

Broadband multiple responses is one of the keys to realize high efficient solar cell^1^,^2^, broadband absorber^3^,^4^, and so on^5^,^6^. There are many ways to broaden the region of interaction frequency, such as using trapezoid units^7^, fractal structure^8^ and photonic quasicrystal^2^,^3^. Photonic quasicrystal (QC) is a class of structures lacking of traditional symmetry, in which blocks are arranged only with long-range order^5^,^9^. The Fourier transformation of the photonic QC gives a set of reciprocal vectors with multi-fold symmetric, which makes the phase matching condition satisfied at various incident frequencies. Correspondingly, these modes could be excited simultaneously under a broadband source, which could be used for increasing the efficiency of energy harvest in solar cell system^1^,^2^,^10^,^11^. The optical response could also be optimized by engineering the density of the spatial frequencies^12^. Plasmonic quasicrystals attract much attention for its various penitential applications^13^. Zi-Lan Deng *et al.* investigate the plasmonic modes in a two dimensional quasicrystalline array of metal nanoparticles with the eigen-decomposition method and two anti-phase ring modes with different polarizations are found to be of high fidelity and high spatial localization^14^. Nanoparticles arranged in both 1D and 2D Fibonacci pattern are also demonstrated to be used in controlling and optimizing the local field enhancement and localization^15^, the in-plane optical mode symmetry^16^, and so on^17^.

In evaluating electromagnetic response of photonic QC, the lacking of short-range order makes periodic boundary conditions no longer be applicable. We must simulate a large enough structure to obtain the long range order property, which makes the traditional numerical methods, including finite-difference time-domain and finite element method, computationally expensive, especially for the 2D photonic QC. Supercell approach is also unable to improve the computational efficiency radically^18^,^19^. In the simulation model of ref. 1, several parameters need to be fitted from the normal incidence spectra of a periodic gold disk arrangement by using the scattering matrix calculations.

As a semi-analysis method, the traditional rigorous coupled wave analysis (RCWA) has advantages in dealing with the photonic crystals, such as 1D grating, 2D square lattice, and even the hexagonal lattice^20^. However, the traditional RCWA needs to be improved to handle the irregular lattice as quasicrystalline structures. In mathematics, quasicrystalline lattice can be obtained by using the cut-and-project method from a higher dimensional space. For example, 1D Fibonacci sequence is generated from the 2D grid to a 1D line and the 2D Penrose tiling is generated from the 5D grid to a 2D plane. It is found that the cut-and-project process does not lose the periodicity of the higher dimensional space. Therefore, the intrinsic periodicity in the quasicrystalline lattice makes the Floquet’s theory still be effective and the RCWA has been used to calculate the 1D Fibonacci grating^19^. Utilization the RCWA on studying the diffraction properties of 2D octagonal quasicrystalline structure is also found^21^,^22^.

In this paper, we study the surface modes excitation in the photonic QC/metal film structure. First, the algorithm of the improved RCWA which can handle the quasicrystalline structures is derived. We choose the quasicrystalline lattice to be the Penrose tiling. Then, we numerically simulate the multiple modes excitation process by the improved new RCWA program. Each of the modes could be identified by comparing with the mode analysis results under the effective media theory. Phase matching condition is still effective, and the excitation intensity is related to the incident polarization. Multiple splitting behavior at the oblique incidence is also found on the spectrum. Modes interactions are also presented by changing the QC constant.

## Method

**Figure 1:**
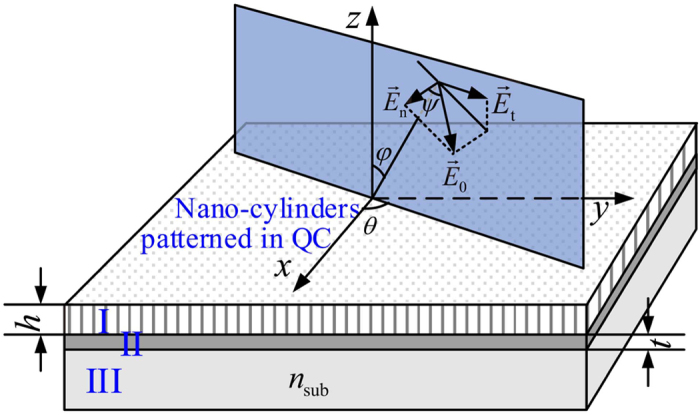
The geometry of the photonic QC/metal film structures and the coordinate configuration. I, II and III are the photonic QC with the cross section as Fig. 2(a), the metal layer and the substrate, respectively. (*θ*, *φ*) represents the incident direction and *ψ* represents the polarization. In this paper, the spectra is calculated with *θ* = *ψ* = *π*/2.

**Figure 2:**
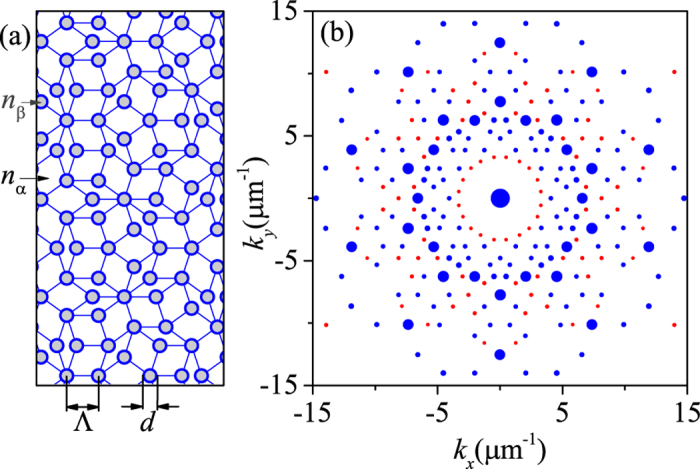
(**a**) The cross section of the photonic QC layer. The gray nano-cylinders of refractive index *n*_*β*_ are patterned in the quasicrystalline lattice with background index *n*_*α*_. (**b**) The Fourier transformation of the quasicrystalline cross section of (**a**) for Λ = 1 μm and *d* = 0.4Λ. The area of each circle is proportional to the spacial spectrum intensity. The circles marked blue are the first 231 reciprocal vectors chosen to be the base vectors.

The proposed photonic QC/metal layer structure on a substrate is as sketched in Figs 1 and 2(a). The quasicrystalline lattice of Penrose tiling is generated by the cut-and-project method from 5D grid to 2D plane^5^,^23^,^24^,^25^. The dielectric nano-cylinders of refractive index *n*_*α*_ are patterned in the quasicrystalline lattice with background index *n*_*β*_. *d* is the diameter of the nano-cylinders and the QC constant Λ is side length of the cells of the Penrose tiling as shown in Fig. 2(a). The thickness of photonic QC and metal film are *h* and *t*, respectively. The structure is placed on the substrate with refractive index of *n*_sub_. (*θ*, *φ*) and *ψ* are used to describe incident angle and incident polarization, respectively.

The RCWA is a semi-analytical method base on the Floquet’s theory. By Fourier transformation, continuous Maxwell equations can be discretized into matrix equations. Then, the eigenmodes can be numerically obtained by solving the eigenvalues and the eigenvectors of the matrix equations. The field in the structure is a linear combination of the eigenmodes. At last, the reflectivity, transmissivity and the field distributions can be obtained by solving the matrix equations establish through the boundary conditions. Two main differences between the traditional RCWA and the improved algorithm in this paper are the choice of the reciprocal vectors and the assembly of the material matrix, which embodies the coupling process between the constituent waves(or Fourier components). The key of the algorithm is to establish a matrix by analyzing the relationship among the reciprocal vectors of the 2D quasicrystalline lattice.

The first step is to discretize Maxwell equations. Without lost of generality, *Q* nano-cylinders distribute in a big circle with radius *R*_0_. The permittivity distribution of the cross section as shown in Fig. 2(a) can be expressed in the convolution form as


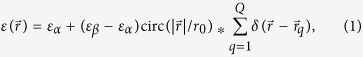


where 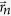 is the center of the *nth* nano-cylinder, “*” represents convolution, 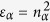, 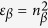, 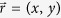 and *r*_0_ is the radius of the nano-cylinder. For the diffraction pattern of any lattice (periodic, aperiodic or quasiperiodic) being a discrete set of points, the Fourier transformation of quasicrystalline lattice should also be a set of Dirac delta functions^5^,^23^. It is possible of expending equation (1) in the form of


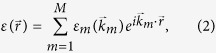


where the base vectors 
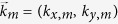
 are the discrete reciprocal vectors of quasicrystalline lattice in the reciprocal space. The expansion coefficients *ε*_*m*_ can be easily derived as


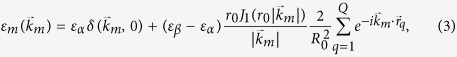


where *J*_1_ is the Bessel function of the first kind. In calculation, we generate *Q* ∼ 1.3 million quasiperiodic points by the cut-and-project method. The result of 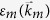 is calculated with *d* = 2*r*_0_ = 0.4Λ = 0.4 μm and has shown in Fig. 2(b). Larger *R*_0_ with large *Q* means the more accurate of the Fourier transformation and *R*_0_ = 500 with *Q* ∼ 1.3 million are enough in the calculation. As expected, the nonzero 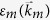 only appear at the terminal points of the base vectors, which are marked in circles. The area of each circle is proportional to the spacial frequency spectrum intensity 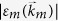.

The fields are expanded into the summary of each constituent waves:


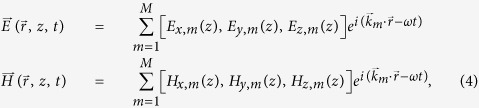


Substitute equation (2) and equation (4) into Maxwell equations, we obtain the matrix equations:


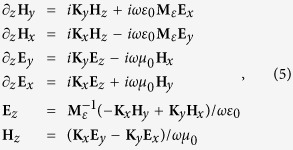


where **E**_*x*_, **E**_*y*_, **E**_*z*_, **H**_*x*_, **H**_*y*_ and **H**_*z*_ are column vectors constructed with elements of *E*_*x*,*m*_, *E*_*y*,*m*_, *E*_*z*,*m*_, *H*_*x*,*m*_, *H*_*y*,*m*_ and *H*_*z*,*m*_, respectively, and **K**_*x*_ and **K**_*y*_ are diagonal matrixes constructed with elements of *k*_*x*,*m*_*δ*_*m*,*n*_ and *k*_*y*,*m*_*δ*_*m*,*n*_, respectively. *δ*_*m*,*n*_ is the Kronecker delta. For arbitrary *m* and *n*, one will always find a 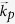 in the ordered set of base vectors satisfying


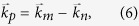


and the element of the material matrix in equation (5) is just 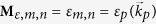. In the traditional RCWA method, equation (6) is equivalent to *p* = *m* − *n*. However, for the quasicrystalline structure, the only way to confirm the order *p* is to traverse the ordered set of base vectors, searching for 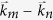.

The properties of the structures are embodied in the material matrix **M**_*ε*_. For homogeneous layers (the layers of air, metal and substrate), **M**_*ε*_ is simply a diagonal matrix. The constituent wave, corresponding to each term of equation (4), propagates independently without coupling. In the photonic QC layer, the nonzero element of **M**_*ε*,*m*,*n*_ denotes the coupling intensity between the constituent waves with 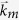 and 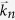.

Equation (5) is an eigenvalue problem essentially. By eliminating **E**_*x*_, **E**_*y*_, **E**_*z*_, **H**_*x*_, **H**_*y*_ and **H**_*z*_, equation (5) can be simplified to a matrix equation:


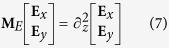


where


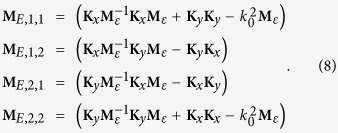


Matrix **M**_*E*_ is divided into four blocks and the two coordinates in the subscript of equation (8) label the position of each block. By solving the eigenvalue problem of equation (7), we obtained the eigenmodes as a combination of the constituent waves of 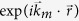. The combination coefficients are just the eigenvector **W**_*m*_ (a column vector with 2M elements and *m* = 1 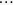 2*M*) with the eigenvalue 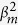 and *β*_*m*_ is the propagation constant of the eigenmodes. Meanwhile, the eigenvectors corresponding to the magnetic field can be obtained as well:


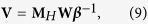


where ***β*** is a diagonal matrix with element *β*_*m*_*δ*_*m*,*n*_, matrix **W** is constructed by **W**_*m*_ and


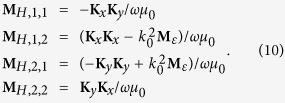


Notice that **M**_*H*_, **W** and ***β*** are all 2M by 2M matrix. The field in layer *n* is the linear combination of the 2M eigenmodes. The combination coefficients express as column vectors 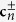 with 2M elements. “+” in the superscript represent the propagation direction of the eigenmode is along the incidence and and “−” corresponds to the opposite. 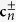 and 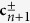 are related by the boundary conditions that the tangential component of **E** and **H** must be continuous at *n*|*n* + 1 interface. For a *N* interfaces system, *N* matrix equations are established:


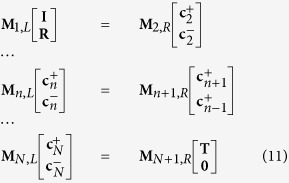


where


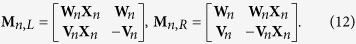


and **I** represent the incident field with the nonzero elements 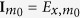 and 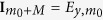 (*m*_0_ is the zero-order diffraction, 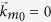). For the normal incidence of TM-polarization, we should set 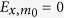 and 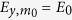. **X**_*n*_ is a diagonal matrix with element and *exp*(*iβ*_*m*_*t*). The field in the structure can be rebuilt with **I**, **R**, **T** and 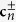, which are solved from equation (11). Taking the reflection field for example, we calculate 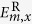 and 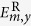 from


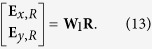


and 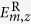 can be calculated from equation (5). The intensity of *m*-order diffraction is


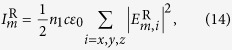


where *n*_1_ is the refractive index region 1. The incident intensity 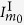 and the transmissive intensity 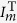 can also be obtained similarly. And the reflectivity and the transmissivity are


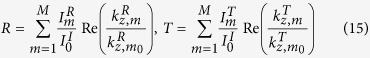


where 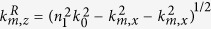 and 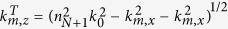. If the plane wave incident at the direction of (*θ*, *φ*) with polarization of *ψ*, two extra steps should be added into the algorithm:


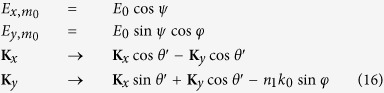


where *θ*′ = *θ* − *π*/2. To improve the numerical stability, we make the same transformation as ref. 26 did, trying to avoid calculating the inverse matrix. For the metallic quasicrystalline structure, although the base mode up to *M* = 421, it is found that the improved RCWA couldn’t be convergent. In the derivation, neither the quasicrystalline lattice is confined to the Penrose tiling, nor the unit is confined to the cylinder. Hence, the algorithm can be directly applied to other quasicrystalline structure with only the modification on equation (3).

## Results

The parameters used in the calculation are *n*_*α*_ = 2.4, *n*_*β*_ = 2.0, *h* = 0.1 μm, *t* = 0.05 μm, and *n*_sub_ = 1.5. The permittivity of silver comes from the Brendel-Bormann model^27^. As is discussed previously, |*ε*_*m*,*n*_| denotes the coupling intensity between constituent waves with wave vector 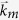 and 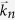. Small |*ε*_*m*,*n*_| means weak energy flow between these two constituent waves. In other words, when 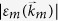 is small enough, the base vector 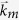 could be ignored with acceptable accuracy. We sort 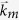 by 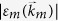 in descending order and take the first *M* of 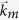
's as the base vectors. It is found that when the cut-off percentage brings down to 5% (i.e., 5%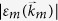), the reflective spectrum becomes stable. It means that we can choose the first 231 of 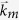
’s, which are marked blue in Fig. 2(b), as the base vectors in calculating the spectrum.

**Figure 3:**
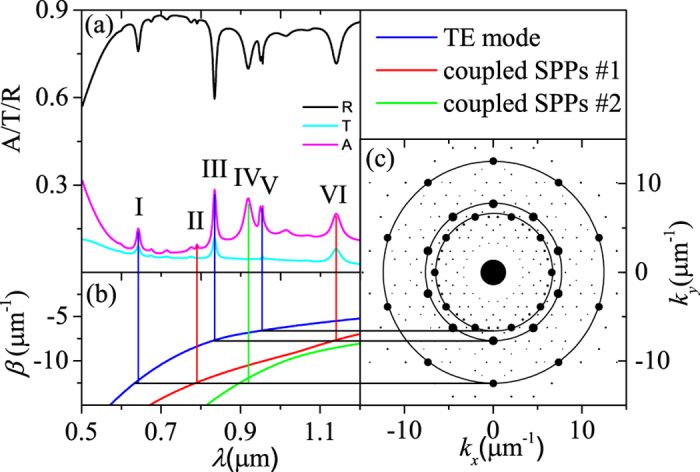
(**a**) The reflection/transmission/absorption spectrum of normal incidence. (**b**) The dispersion relations from the mode analysis of the air/effective media/metal film/substrate structure. The blue line is TE mode. The red and the green line are the coupled SPPs along the upper/lower metal surface. (**c**) The reciprocal vectors in reciprocal space.

Figure 3(a) shows the reflection/transmission/absorption spectrum for TM-polarized normal incidence. The complex spectrum structure means that a serious of surface waves are excited in the region of *λ* ∈ (0.5 μm, 1.2 μm).

To identify these reflection valleys on the spectrum, we analyze both the TE and TM modes in the multilayer structure. Under the effective medium theory, the photonic QC is simplified to a homogeneous layer with effective index *n*_eff_ = (1 − *f*)*n*_*α*_ + *fn*_*β*_ ≈ 2.06, where *f* ≡ *S*_*α*_/(*S*_*α*_ + *S*_*β*_), *S*_*α*_ represent the sum of the areas of the nano-cylinders and *S*_*β*_ represent the area of the rest. The dispersion relations, including a TE waveguide (blue) and two surface plasmon polaritons(SPPs, red and green), are plotted in Fig. 3(b). The repulsion of two SPPs branches (red and green) means that strong coupling exists between the SPPs on the upper and lower interface.

In the excitation process of 2D modes, the phase matching condition reads


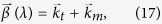


where 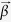 is the wave vector of the excitation mode, 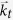 is the tangential component of incident wave vector projecting to the *xy* plane and 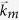 is the reciprocal vector of QC. Because of the internal 10-fold symmetry, when 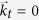 at normal incidence, each of ten reciprocal vectors on the same circle should satisfy equation (17) simultaneously. It means that each reflection valley in Fig. 3(a) corresponds to the ten surface waves with same 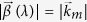 but different propagation directions. These degenerate modes will be separates at oblique incidence. The connection between Fig. 3(a,b,c) are established by the vertical and horizon color lines. Mode I, III and V correspond to the same dispersion relation of blue curve in Fig. 3(b) but three different circles with three radiuses of 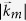 in Fig. 3(c). Because the dispersion relation of blue curve refers to the TE mode, these three modes are determined to be the TE modes guided by the photonic QC layer. Mode II, IV and VI correspond to the dispersion relations of red and green curves in Fig. 3(b). Because the dispersion relations of red and green curves refer to SPPs, these three modes are determined to be the SPPs on the upper and lower metal surface.

**Figure 4:**
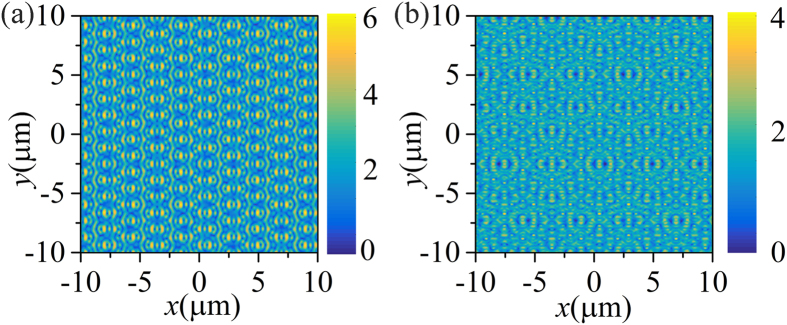
The field distributions of |*E*| at upper surface of metal film for normal incidence. (**a**,**b**) correspond to mode III at *λ* = 0.833 μm and mode IV at *λ* = 0.922 μm, respectively. (see Fig. 3).

Taking mode III at *λ* = 0.833 μm and mode IV at *λ* = 0.922 μm as examples, we plot the field distribution of |*E*| at upper surface of metal film in Fig. 4(a,b), respectively. The field distributions for normal incidence just behave as the standing wave with nodes, which means the surface waves at opposite directions are excited at the same time. It is also found that the main propagation directions of mode III and IV are different, which result from the polarization differences between TE mode and SPPs. It will be discussed in detail in the later sections.

**Figure 5:**
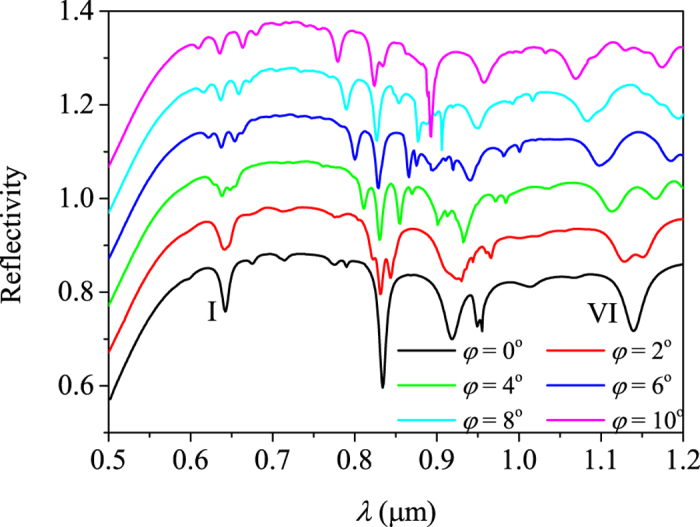
The reflection spectra with incident angle *φ* = 0° ∼ 10° (*ψ* = *θ* = *π*/2). The vertical axis corresponds to the black solid line. Others shift up 0.1 by tune.

For oblique incidence of *φ* ≠ 0, the spectrum shows a complex splitting behavior as plotted in Fig. 5. When *φ* ≠ 00, although the 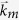
’s on the same circle are equal in magnitude, the nonzero 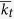 makes 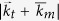
’s no longer degenerate. For the 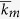
’s on the small circle in Fig. 3(c), six sub-reflection-valleys should appear in theory, and for the 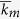
’s on the middle and large circles, five sub-reflection-valleys should appear. Yet, not all of these sub-modes are found in Fig. 5. It is difficult to analyze mode II ~ V for the complex superposition. Therefore, we concentrate on mode I and VI. They obviously don’t split into five sub-modes as the analysis. Especially for mode VI, the reflection valley performs like a double split behaviour. We attribute the sub-mode absence to the polarization.

Firstly, let’s focus on mode I. As a TE-polarized mode, the magnetic field is on the plane determined by *z* axis and 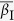, and the electric field is perpendicular to this plane. On the other hand, as TM-polarized incidence, the electric field is on *yz* plane and the magnetic field is along *x* axis. We infer that the excitation intensity is determined by the angle between incident field and mode field. The sub-mode with 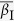 along *x* axis has electric field along *y* axis, just matching the electric field of incidence. It means that the sub-modes with 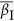 close to the *x* axis should be strongly excited. The depth of the reflection valley is proportional to the excitation intensity of corresponding mode. Among the sub-reflection-valleys splitting from mode I, the valley with maximum depth should appear at the center just as displayed in Fig. 5.

Next, we focus on mode VI. As a TM-polarized mode, the direction of field is just opposite to the TE-polarized mode, i.e., the magnetic field is perpendicular to the plane determined by *z* axis and 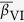. Base on the same inference, the splitting modes with 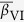 close to the *y* axis should have the consistent field direction with the incidence and should be strongly excited. Hence, the deepest valley should appear at two sides as shown in Fig. 5.

**Figure 6:**
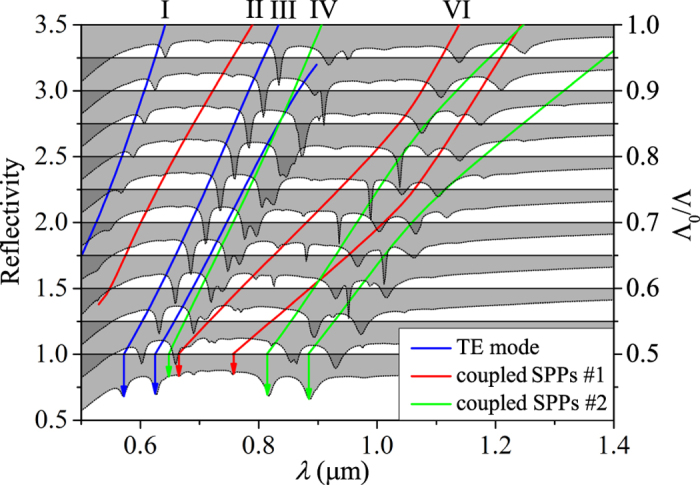
The reflection spectra of normal incidence changing with the QC constant Λ (Λ_0_ = 1 μm). The blue, red, and green lines are the dispersion relation from the mode analysis (dispersion relation *β*(*λ*) with equation (18)). The vertical axis corresponds to Λ = 2 μm(Λ_0_/Λ = 0.5). Others shift up Δ*R* = 0.25 for the spectra by tune, corresponding to Δ(Λ_0_/Λ) = 0.5 as right labels shown.

It is noteworthy that the SPPs of mode II, IV and VI in Fig. 3(a) are excited with the different intensities. That is, mode II is weakly excited, while mode IV and VI are strong. To understand this difference, we plot the reflectivity spectra changing with the QC constant Λ in Fig. 6. According to equation (17) and mode analysis, each value of 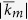 corresponds to three surface modes (seen as a group). There are three main values of 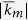 for the condition of *d* = 0.4Λ. Therefore, nine dispersion relations in three group should be found as the color solid lines in Fig. 6:


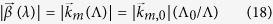



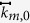
 is the reciprocal vector when Λ_0_ = 1 μm and the three main 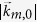
's in Fig. 3(c) are 2*π*sec(*π*/10) μm^−1^, 8*π*sin (*π*/10) μm^−1^ and 4*π* μm^−1^, corresponding to the radiuses of three circles. The blue lines are the dispersion relations of the TE mode. The strong coupling between SPPs on the upper and lower metal surface leads to the break off of the SPPs dispersion relations. The former part of the green lines together with the latter part of the red lines are the SPPs dispersion relations along the upper metal surface, and the former part of the red lines together with the latter part of the green lines are the SPPs dispersion relations along the lower metal surface. By comparing dispersion relations with the reflection spectra, TE modes would be always strongly excited until the cut-off wavelength *λ* ∼ 0.9 μm, when the thickness of quasicrystalline layer is too thin to afford TE mode. It also found that the SPPs on the upper metal surface are easy to be excited, while the SPPs on the lower metal surface are difficult to. Similar phenomenon could also be found in the asymmetric metal/dielectric corrugated structures^28^. The coupling process doesn’t exist between groups because of the magnitude of 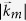 being different. Back to the spectrum in Fig. 3(a), mode II at *λ* = 0.789 μm is the SPPs on the lower metal surface, which is difficult to be excited. The other two mode, IV and VI, belong to the SPPs on the upper metal surface, which could be strongly excited.

## Summary

We have numerically studied the broadband multiple responses of the surface modes in 2D photonic QC/metal structures. The 2D photonic QC here refers to the the dielectric cylinders patterned in Penrose tiling. We also improve the RCWA method so that it can handle quasicrystalline lattice. When the cut-off percentage of reciprocal vectors is chosen to be 5% (i.e., 231 reciprocal vectors are chosen to be the base vectors), the calculation shows an accurate enough results with acceptable efficiency.

Six main responses on the normal incidence spectrum, which correspond to three SPPs modes and three TE modes, are observed. Each mode multiply splits to 2∼4 sub-modes at oblique incidence. If the incident wave is TM-polarized, the sub-modes have the maximum response on the center for TE mode and on two sides for SPPs. If we change the polarization of incidence, the position of the maximum response would change too.

The excitation frequency of the proposed structure is determined by the phase matching condition. However, not all of the excitation frequencies satisfying phase matching condition can be found on the reflection spectrum and the excitation intensities of some modes are too weak to be observed on the spectrum. On one hand, the SPPs on the lower metal surface is unable to be excited strongly. The main indication of its existence is the break-off of the dispersion relations of the SPPs on the upper surface, which is caused by the coupling between the two SPPs modes. The coupling between other modes are not found. On the other hand, the excitation intensity also depends on the angle between incident field and mode field. It means that some sub-modes at oblique incidence might be too weak to present on the spectrum.

## Additional Information

**How to cite this article**: Yuan, H. *et al.* Broadband multiple responses of surface modes in quasicrystalline plasmonic structure. *Sci. Rep.* **6**, 30818; doi: 10.1038/srep30818 (2016).
